# Environmental Factors and Interactions with Mycobiota of Grain and Grapes: Effects on Growth, Deoxynivalenol and Ochratoxin Production by *Fusarium culmorum* and *Aspergillus carbonarius*

**DOI:** 10.3390/toxins2030353

**Published:** 2010-03-18

**Authors:** Naresh Magan, David Aldred, Russell Hope, David Mitchell

**Affiliations:** Applied Mycology Group, Cranfield Health, Cranfield University, Bedford MK43 0AL, UK; Email: d.aldred@cranfield.ac.uk (D.A.); russell.hope@hpa.org.uk (R.H.); david.mitchell7@spcorp.com (D.M.)

**Keywords:** fungal interactions, environmental factors, mycotoxins, interspecific interactions

## Abstract

Mycotoxigenic fungi colonizing food matrices are inevitably competing with a wide range of other resident fungi. The outcomes of these interactions are influenced by the prevailing environmental conditions and the competing species. We have evaluated the competitiveness of *F. culmorum* and *A. carbonarius* in the grain and grape food chain for their *in vitro* and *in situ* dominance in the presence of other fungi, and the effect that such interactions have on colony interactions, growth and deoxynivalenol (DON) and ochratoxin A (OTA) production. The Index of Dominance shows that changes in water activity (a_w_) and temperature affect the competitiveness of *F. culmorum* and *A. carbonarius* against up to nine different fungi. Growth of both mycotoxigenic species was sometimes inhibited by the presence of other competing fungi. For example, *A. niger* uniseriate and biseriate species decreased growth of *A. carbonarius*, while *Aureobasidium pullulans* and *Cladosporium* species stimulated growth. Similar changes were observed when *F. graminearum* was interacting with other grain fungi such as *Alternaria alternata*, *Cladopsorium herbarum* and *Epicoccum nigrum*. The impact on DON and OTA production was very different. For *F. culmorum*, the presence of other species often inhibited DON production over a range of environmental conditions. For *A. carbonarius*, on a grape-based medium, the presence of certain species resulted in a significant stimulation of OTA production. However, this was influenced by both temperature and a_w_ level. This suggests that the final mycotoxin concentrations observed in food matrices may be due to complex interactions between species and the environmental history of the samples analyzed.

## 1. Introduction

Cereal grain during ripening as well as grape development represent food ecosystems that are colonized by a mixed mycobiota, which are influenced by abiotic factors such as prevailing temperature and relative humidity, especially at a microclimate level [1,2]. Thus, the fungi colonizing these ecological niches will interact with each other as they compete to utilize the available nutrients. The level of niche overlap or competitiveness of individual species may be related to environmental tolerance and may also be related to rates of germination and growth, production of extracellular enzymes and secondary metabolites such as mycotoxin production to provide a competitive edge [3,4]. 

Magan and Lacey [5] demonstrated that changes in environment can significantly impact on fungal interactions and alter the competitiveness of individual spoilage species, based on studies on wheat-based media. Subsequent work with a range of spoilage mycotoxigenic fungi has supported the impact that interacting environmental factors have on interactions and dominance of specific species in different stored food matrices [6,7,8,9]. Changes in the environment or other stress factors such as fungicide applications or application of aliphatic acid-based preservatives may also lead to one species having an advantage over competitors. This has been shown in field trials with mycotoxigenic *Fusarium* species and interactions with non-mycotoxigenic plant pathogens of wheat such as *Microdochium nivale* [10,11]. However, very little information is available on the effect of interactions between deoxynivalenol (DON) producing *Fusaria*, other *Fusarium* species and phyllosphere mycoflora species under different environmental conditions.

The interaction of spoilage fungi when studied *in vitro* can be macroscopically scored [5] by observing the macro and microscopic interactions and giving each interacting species numerical scores to represent categories or interaction type. Thus, mutual intermingling (1-1) was given a lower score than mutual interactions (2-2, 3-3) and dominance by one species over another (4-0, 5-0). The scores for each species can be added to obtain an overall Index of Dominance (I_D_). This score can then be compared to see variations under different environmental conditions. Interaction and competition between *Aspergillus ochraceus* (=*A. westerdijkiae*) and other species has been shown to have a marked influence on ochratoxin (OTA) production [7,12]. 

The objective of this study was to examine the effect of a_w_ and temperature on inter-specific interactions between (a) *F. culmorum* and other cereal fungi, and (b) *A. carbonarius* and related grape colonizing fungi on growth and DON and OTA production.

## 2. Results

### 2.1. Effects of interactions between *Fusarium culmorum* and other mycobiota on growth and DON production on wheat-based matrices

Table 1 shows the interactions on wheat grain at 15 and 25 °C and both 0.995 and 0.955 a_w_. *F. graminearum* and *F. poae* were dominant over *F. culmorum* at most a_w_ levels and the two temperatures. Where the other *Fusaria* were not dominant over *F. culmorum*, mutual inhibition on contact type interactions occurred. Other phyllosphere fungi were mostly dominated by *F. culmorum* in all the conditions tested. The sum of the I_D_ scores shows that *F. culmorum* was overall more competitive than the other species, and thus dominant under the conditions examined on wheat grain.

**Table 1:** Interactions and Index of Dominance scores for interactions of *Fusarium culmorum* with other phyllosphere fungi on wheat grain at different water activity and temperature conditions. In all cases, the first score is for *F. culmorum*. Key to fungi: *F.*, *Fusarium; A.*, *Alternaria; M.*, *Microdochium*, *P.*, *Penicillium*.

**Species**	**Temperature (°C)**					
	**15**			**25**		
	**0.995**	**0.955**	**I_D_**	**0.995**	**0.955**	**I_D_**
***F. graminearum***	0/4	0/4	**0/8**	0/4	0/4	**0/8**
***F. poae***	2/2	0/4	**2/6**	1/1	2/2	**3/3**
***A. tenuissima***	4/0	4/0	**8/0**	4/0	4/0	**8/0**
***C. herbarum***	4/0	4/0	**8/0**	4/0	4/0	**8/0**
***M. nivale***	2/2	4/0	**6/2**	4/0	4/0	**8/0**
***M. nivale var majus***	2/2	0/4	**2/6**	4/0	4/0	**8/0**
***P. verrucosum***	4/0	2/2	**6/2**	2/2	4/0	**6/2**
**Total I_D_**	**18/10**	**14/14**	**32/14**	**19/7**	**22/6**	**41/13**

**Figure 1:**
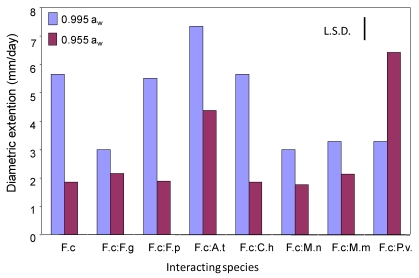
Comparison of the effect of interactions on relative growth rate of *F. culmorum* when in dual culture with different wheat grain fungi at 0.995 and 0.955 water activity at 25 °C on wheat grain. Key to fungi: F.c, *F. culmorum*; F.g, *F. graminearum*; F.p, *F. poae*; A.t, *A. tenuissima*; C.h, *C. herbarum*; M.n, *M. nivale*; M.m, *M. nivale* var *majus*; P.v, *P. verrucosum*. L.S.D., Least significant difference at P = 0.05.

Figure 1 shows the effect of such interactions on the relative growth of *F. culmorum* on wheat grain. Growth rates were unaffected by interactions with *C. herbarum*, *F. graminearum*, *F. poae*, and *M. nivale* and *M. nivale* var *majus* at 0.995 and 25 °C. At 0.995 a_w_ and this temperature, all the fungi inhibited growth of *F. Culmorum*, except for *C. herbarum* and *P. verrucosum*. However, at 15 °C, growth of *F. culmorum* was stimulated by interactions in dual culture with *A. tenuissima*, *P. verrucosum*, *C. herbarum*, and the *Microdochium* species. 

### 2.2. Effect of interactions between *F. culmorum* and other grain mycobiota on deoxynivalenol production on wheat grain

**Figure 2:**
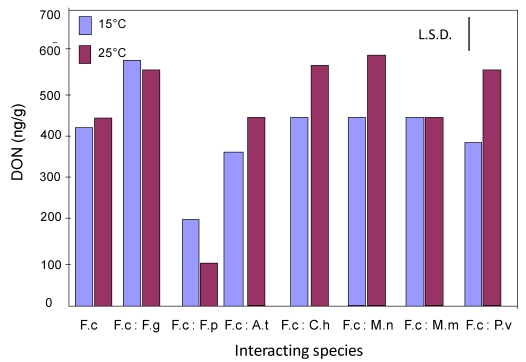
Comparison of the amount of DON produced by *F. culmorum* alone and when interacting with different wheat grain fungi at 0.955 water activity at 15 and 25 °C. Key to fungi: F.c, *F. culmorum*; F.g, *F. graminearum*; F.p, *F. poae*; A.t, *A. tenuissima*; C.h, *C. herbarum*; M.n, *M. nivale*; M.m, *M. nivale* var *majus*; P.v, *P. verrucosum*. L.S.D., Least significant difference at P = 0.05.

Figure 2 shows the effect of interactions with other species in dual culture on the production of DON at 15 and 25 °C at 0.955 a_w_. It is shown that in the presence of *M. Nivale*, there was stimulation of the production of DON by *F. culmorum*. In the presence of some species, DON production was inhibited. Table 2 summarizes, based on the statistical analyses, whether DON was stimulated or inhibited or whether there was no effect due to interactions on wheat grain. 

**Table 2:** Effect of interactions between *F. culmorum* and other grain fungi on DON production at different water activity and temperature conditions on wheat. Key: NS, not significantly different from control; ↑↓, significant increase or decrease in DON production relative to the control. Key to fungi: F.c, *F. culmorum*; F.g, *F. graminearum*; F.p, *F. poae*; A.t, *A. tenuissima*; C.h, *C. herbarum*; M.n, *M. nivale*; M.m, *M. nivale* var *majus*; P.v, *P. verrucosum*.

	**Temperature (°C)**			
	**15**		**25**	
**Water activity**	**0.995**	**0.955**	**0.995**	**0.955**
F.c:F.g	NS	↑	↓	↑
F.c:F.p	NS	NS	↓	NS
F.c:A.t	NS	NS	↓	NS
F.c:C.h	NS	NS	↓	↑
F.c:M.n	NS	NS	↓	↑
F.c:M.m	NS	NS	↓	NS
F.c:P.v	NS	NS	↓	↑

### 2.3. Effects of interactions between *A. carbonarius* and other mycobiota on growth and OTA production on grape-based matrices

The effect of a_w_ and temperature interactions on relative competitiveness of *A. carbonarius* against different fungi are shown in Table 3. The relative total I_D_ values under the different conditions are also shown. Overall, three different interaction types were found in the study. At 30 °C, *A. carbonarius* generally dominated all the other fungi, scoring 4-0. The exceptions were the other *Aspergillus* section *Nigri* species and *E. nigrum*. At 20 °C, *A. carbonarius* was only able to dominate the pink yeast. Against all other fungi *A. carbonarius* was mutually antagonistic using contact scoring (2-2), whilst with *E. nigrum* it was mutually antagonistic at a distance, scoring 3-3.

The growth rates varied with temperature and a_w_ level of treatment. When comparing the growth rate of *A. carbonarius* in the absence of competitors against growth after interactions in dual culture, there was a slight inhibition of the area of colonization. Table 4 shows the relative stimulation or inhibition of growth by competitors when compared to *A. carbonarius* alone. This shows that depending on the interacting species, there was an effect on growth of the mycotoxigenic OTA producing species. 

**Table 3:** Interaction and Index of Dominance (I_D_) scores for *A. carbonarius* *versus* various vineyard fungi on a synthetic grape juice medium at two water activity levels, incubated at three temperatures.

	**30 °C**			**25 °C**			**20 °C**		
**a_w_/species**	**0.98**	**0.95**	**0.93**	**0.98**	**0.95**	**0.93**	**0.98**	**0.95**	**0.93**
*Cladosporium* species	4/0	4/0	4/0	4/0	4/0	2/2	4/0	2/2	2/2
*Epicoccum nigrum*	2/2	2/2	2/2	3/3	3/3	3/3	3/3	3/3	3/3
*Aureobasidium pullulans*	4/0	4/0	4/0	4/0	4/0	2/2	2/2	2/2	1/1
*Aspergillus* section *Nigri biseriate*	2/2	2/2	2/2	2/2	2/2	2/2	2/2	2/2	2/2
*Botrytis cinerea*	4/0	4/0	4/0	4/0	4/0	4/0	2/2	2/2	2/2
*Alternaria alternaria*	4/0	4/0	4/0	4/0	4/0	2/2	2/2	2/2	2/2
*Aspergillus* section *Nigri uniseriate*	2/2	2/2	2/2	2/2	3/3	2/2	3/3	3/3	2/2
*Phoma* species	4/0	4/0	4/0	4/0	4/0	2/2	2/2	2/2	2/2
Pink yeast	4/0	4/0	4/0	4/0	4/0	4/0	4/0	4/0	4/0
**I_D_**	**30/6**	**30/6**	**30/6**	**31/7**	**32/8**	**19/15**	**24/16**	**22/18**	**20/16**

**Table 4:** Relative stimulation or inhibition of growth of *A. carbonarius* versus various vineyard mycoflora in relation to water activity and three temperatures. + indicates a stimulation in growth and - a decrease in growth rate compared to *A. carbonarius* grown alone.

	**30 °C**			**25 °C**			**20 °C**		
**a_w_/species**	**0.987**	**0.95**	**0.93**	**0.987**	**0.95**	**0.93**	**0.987**	**0.95**	**0.93**
*Aspergillus carbonarius*	5.1	4.8	4.0	5.0	4.5	3.5	3.9	2.8	2.2
*Cladosporium* species	5.4	5.8^+^	6.6^+^	5.2	5.0^+^	3.2	3.5	1.9^-^	1.2^-^
*Epicoccum nigrum*	N/A	N/A	N/A	5.7^+^	4.4	2.3^-^	5.9^+^	3.4^+^	2.5
*Aureobasidium pullulans*	5.9^+^	6.3^+^	4.7^+^	3.8^-^	5.0^+^	3.7	2.6^-^	1.6^-^	2.4
*Aspergillus* section *Nigri biseriate*	2.2^-^	2.2^-^	2.4^-^	3.6^-^	2.2^-^	1.5^-^	3.6	2.5	0.9^-^
*Botrytis cinerea*	7.1^+^	7.6^+^	4.2	4.8	4.4	3.6	2.9^-^	2.6	1.2^-^
*Alternaria alternaria*	6.5^+^	5.8^+^	4.1	5.3	4.6	4.1^+^	3.6	3.8^+^	1.9
*Aspergillus* section *Nigri uniseriate*	4.5^-^	3.4^-^	4.0	4.9	3.3^-^	2.7^-^	3.6	2.2^-^	0.9^-^
*Phoma* species	5.2	4.9	6.4^+^	4.7	3.5^-^	2.6^-^	3.6	2.2^-^	1.0^-^
Pink yeast	6.7^+^	5.8^+^	4.2	4.9	4.6	1.8^-^	3.7	2.6	1.4^-^

### 2.4. Ochratoxin A production by *A. carbonarius* when grown against other common grape fungi

**Figure 3:**
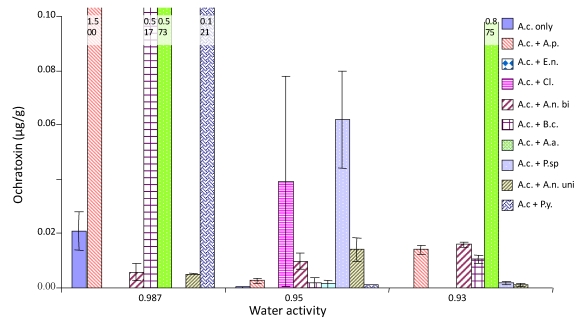
Ochratoxin production by *A. carbonarius* at 20 °C when grown against grape vineyard fungi. Grown on Synthetic grape juice medium at 0.93-0.987 a_w_ levels. Bars indicate standard error of the mean. Key to species A.c; *A. carbonarius*, A.p; *A. pullulans*,E.n; *E. nigrum*,Cl; *Cladosporium* species,A.n bi; *A*. section *Nigri biseriate*,B.c; *B*. *cinerea*, A.a; *A*. *alternata*, P.sp; *Phoma* species, A.n uni; A. section *Nigri uniseriate*, p.y; Pink yeast.

**Figure 4:**
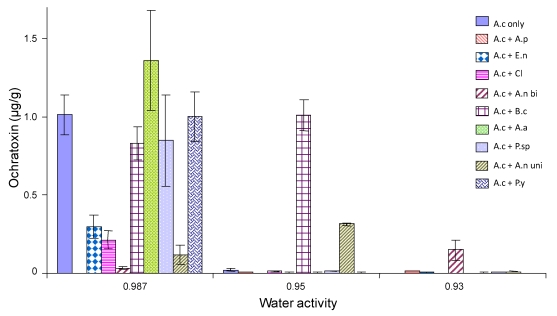
Ochratoxin production by *A. carbonarius* at 25 °C when grown against grape vineyard fungi. Grown on Synthetic grape juice medium at 0.93-0.987 a_w_ levels. Bars indicate standard error of the means. Key to species as for Figure 3.

**Figure 5:**
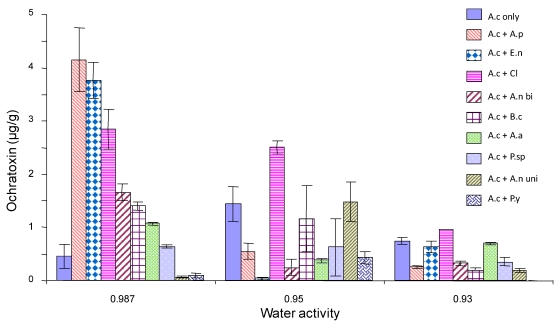
Ochratoxin production by *A. carbonarius* at 30 °C when grown against grape vineyard fungi. Grown on Synthetic grape juice medium at 0.93-0.987 a_w_ levels. Bars indicate standard error of the means. Key to species as for Figure 3.

Figure 3, Figure 4 and Figure 5 show the effect of interactions between *A. carbonarius* and other fungi on OTA production under different environmental conditions. OTA production was greatest at 20 °C and 0.987 a_w_ with stimulation occurring when grown against most other species (Figure 3). OTA production decreased when the a_w_ was decreased (drier conditions) and with increasing temperature. The lowest OTA production was at 30 °C and 0.93 a_w_. In general, there was a stimulation of OTA production at 0.987 a_w._ However, OTA production was reduced at 0.93 and 0.95 a_w_ when compared with that produced by *A. carbonarius* alone. Interaction with *B*. *cinerea* consistently resulted in increased OTA production when compared to the control. Competition with the pink yeast (*Sporobolomyces* species) and *Aspergillus* section *Nigri* (uniseriate) also resulted in reduced OTA produced by *A. carbonarius* than when grown alone.

## 3. Discussion

### 3.1. *F. culmorum* and DON production

It is interesting that *F. culmorum* was often not able to achieve dominance over *F. graminearum*, regardless of the environmental conditions, and they were mutually antagonistic to each other at 0.955 a_w_. This supports published work reported on FEB, which has shown dominance of *F. graminearum* inoculum and infection in the UK and indeed in Europe [13,14]. The interactions also had a variable effect on colonization by *F. culmorum*. In some cases, growth was significantly increased (*F. culmorum* versus *A. tenuissima*) at 25 °C and both 0.995 and 0.955 a_w_. In other cases, growth was significantly reduced (*F. culmorum* versus *F. graminearum*, 25 °C and 0.995 a_w_). However, changes in growth rate did not seem to correlate with changes in interaction type. For instance, *F. culmorum* was dominant in contact with *A. tenuissima* in all conditions assayed at 25 °C with growth was significantly stimulated by this interaction, whereas at 15 °C there was no difference from the control. 

Earlier interaction studies by Marin *et al.* [8] examined *Fusarium* species on maize and found that there was no correlation between populations of *F. verticillioides* and four other interacting species and interaction scores. Studies by Lee and Magan [7] found that *A. ochraceus* growth rates were reduced by the presence of other fungi on maize and no stimulation observed, regardless of temperature and a_w_ levels tested. This suggests that growth rate alone does not determine competitiveness of a specific species *per se*. Recent studies have suggested that carbon nutritional patterns may also play a significant role in the level of niche occupation or niche exclusion, and that this is further influenced by the changing environmental factors [4,9]. 

Practically no studies have been carried out to evaluate the impact of interactions on DON production by *F. culmorum*. Changes in DON concentrations occurring while interactions remain the same may indicate a change in combative strategy by the fungi in response to environmental stress. For example, *F. graminearum* was able to dominate *F. culmorum* on wheat grain at 0.995 and 0.955 a_w_. However, at the higher a_w_ level, DON was significantly reduced, whereas at 0.955 a_w_ DON levels were increased. As they both produce DON it is possible that under water stress they both increase production of DON resulting in the increased concentrations observed. This could indicate a physiological change in response to ecological pressures, even though the interaction outcomes were macroscopically the same. However, generally it has been suggested that when some fungi are in close proximity to each other, mycotoxin production is stimulated; this is thought to be because the fungi are trying to pre-emptively exclude other competitors [3,4]. 

For example, Ramakrishna *et al.* [12] found that T-2 toxin production by *Fusarium sporotrichioides* was generally inhibited when grown in dual culture with *Aspergillus flavus*, *P. verrucosum* and *Hyphopichia anomala* on barley grain. However, in the present study some stimulation of toxin production was observed under some interacting environmental conditions.

### 3.2. *A. carbonarius* and OTA production

Very few studies have examined the effect the competitiveness of *A. carbonarius* against other mycobiota of grapes. The effects of a_w_ and temperature suggest that at 30 °C *A. carbonarius* is very competitive and able to outcompete and dominate all the non-*Aspergillus* species, except *E. nigrum*. *A. carbonarius* has an optimum growth temperature of around 30 °C [15] and thus would certainly be able to colonize grape-based matrices quickly over a range of a_w_ conditions. The competitiveness of *A. carbonarius* in these conditions was also supported by the I_D _results and the relative influence on growth rates. At lower temperature conditions, which are sub-optimal for growth (20-25 °C), *A. carbonarius* was less competitive, except against the pink yeast strain. Under most conditions there was mutual antagonism on contact. Both *E. nigrum* and *Aspergillus* section *nigri* (uniseriate) showed signs of being antagonistic at a distance suggesting the influence of secondary metabolites. These two species have a wide optimum growth range, and in the case of *E. nigrum*, a higher optimal a_w _as well [5]. However, it can compete effectively as it is also a biocontrol agent of some preharvest pathogens and produces a wide range of pigments and secondary metabolites [16].

With regard to OTA production, we had nine different interacting species and nine different environmental conditions. This resulted in outcomes from interactions being both stimulatory and inhibitory to OTA production by *A. carbonarius* being observed. At 30 °C and with freely available water (0.98 a_w_), OTA production was decreased in the presence of all competitive fungi except for *A. pullulans*, *A. alternata* and the pink yeast. These were the species which stimulated growth of *A. carbonarius*. At 20 °C and 0.98 a_w_ there was a stimulation of OTA production by *A. carbonarius* when grown in the presence of all species except the pink yeast and the *Aspergillus* section *Nigri* (uniseriate) species. As a_w_ was reduced to 0.95 a_w_ at this temperature OTA production was suppressed by interaction with all species except *Cladosporium* species. Previous ecological studies suggest that 20-25 °C and 0.95 a_w_ are optimum conditions for OTA production [15]. This is very different from those for optimum growth which are 30 °C and 0.98 a_w_. Thus, under a_w_ stress, more OTA may be produced as a defence reaction against competitors to maintain colonization/occupation of the niche. Of course at 0.95 a_w_ most of the competing fungi (phyllosphere species) are under some stress with reduced growth. Thus the effect of competition in relation to growth and OTA production may be quite complex. The ability of *B. cinerea* to inhibit OTA production is interesting as it is a saprophyte which normally infects senescing leaves and plant material. It colonizes damaged grapes under high humidity conditions and is able to produce high amounts of hydrolytic enzymes. It may be that this enables *B. cinerea* to compete effectively with *A. carbonarius* under some conditions and prevent the biosynthesis of OTA. This again suggests that the interactions between fungi are complex and the influence of changing environmental conditions will influence not only the outcome but the role of mycotoxins in competition.

## 4. Experimental Section

### 4.1. Fungal isolates used in this study

Table 5 lists the fungi used in interaction studies related to (a) grain (b) grape-based ecosystems.

**Table 5:** List of fungi used in interaction studies. Those highlighted in bold were the key mycotoxigenic fungi considered in interaction studies. DON, deoxynivalenol; OTA, ochratoxin. Key to sources of fungi: CC, Rothamsted Research, UK.; IMI, International Mycological Institute; IBT, Technical University, Denmark; SVF, Lleida University, Spain; N/M, Harper Adams University College, UK.

**Ecosystem type**	**Strain number**	**Cereals **	**Grapes**
***Fusarium culmorum* (DON)**	CC171	+	-
***Fusarium graminearum* (DON)**	CC175	+	-
***Fusarium poae***	CC179	+	-
***Aspergillus carbonarius* (OTA)**	IMI388653	-	+
*Aspergillus carbonarius* (uniseriate)	IMI388862	-	+
*Aspergillus niger* (biseriate)	IMI388550	-	+
***Penicillium verrucosum* (OTA)**	IBT2266	-	+
*Microdochium nivale*	18/1/N	*+*	*-*
*Microdochium nivale* var *majus*	1/1M	*+*	*-*
*Epicoccum nigrum*	SVF01	+	+
*Cladosporium herbarum*	IBT7961	+	+
*Alternaria alternata*	IBT8320	+	+
*Aureobasidium pullulans*	SVF02	-	+
*Botrytis cinerea*	SVF03	-	+
Pink yeast (*Sporobolomyces* species)	SVF06	-	+
*Phoma* species	SVF05	-	+

### 4.2. Media used

*Wheat-based studies:* For wheat mycobiota interaction studies and effect on DON production, experiments were initially conducted on a 2% milled wheat agar medium modified with glycerol to the required a_w_ levels (0.995, 0.98, 0.955) at 15 and 25 °C. Subsequent studies and those reported in this paper were carried out on layers of wheat grain modified to the required a_w_ levels by reference to a moisture adsorption curve at 0.995 and 0.955. After equilibration of irradiated (12 kGys) grain, the single layers were placed in sterile 9 cm Petri plates and placed in polyethylene containers containing glycerol/water solutions to maintain the equilibrium relative humidity at the target level. The layers of grain were inoculated with the test fungi using agar plugs or spore suspensions 4 cm apart. The growth of each species was measured over a period of 10-14 days and the type of interactions determined. The experiments were carried out with three replicates per treatment and performed twice.

*Grape-based studies:* Studies were carried out on a synthetic grape-based medium (SGM) representative of mid-veraison [15]. This consisted of D(+) glucose 70g, D(-) fructose 30g, L(-) tartaric acid 7g, L(-) malic acid 10 g, (NH_4_)_2_HPO_4_ 0.67 g KH_2_PO_4_ 0.67 g, MgSO_4 _7 H_2_O 1.5 g NaCl 0.15 g CaCl_2_ 0.15 g CuCl_2_ 0.0015 g, FeSO_4 _7 H_2_O 0.021 g, ZnSO_4 _7 H_2_O 0.0075 g, (+) Catechin hydrate 0.05 g, agar 25 g in a litre of medium. This was adjusted with 2 M KOH to pH 4.0-4.2. The studies were carried out at 0.98 and 0.95 a_w_,at 25 and 30 °C. Seven day old colonies were used to prepare spore suspensions of (1 × 10^6^) of *A. carbonarius*, *B. cinerea*, *Phoma sp*, pink yeast, white yeast, *Aspergillus* section *Nigri* biseriate and uniseriate. Due to problems in harvesting spores of *E. nigrum and A. alternate*, mycelium from the growing edge of colonies was included in the spore suspension. SGM plates were then inoculated using a 1 μL calibrated loop, with *A. carbonarius* and one of the other species inoculated approximately 4 cm apart. Controls were inoculated centrally using the 1 μL loop. Each treatment was replicated three times and plates of the same a_w_ were sealed in plastic bags. The plates were incubated for 15 days and analyzed for OTA production. All experiments were carried out twice with three replicates on each occasion.

### 4.3. Measurements of growth rates and interaction scores

Colony diameter was measured by taking two measurements at right angles to each other during the incubation period to calculate a growth rate by linear regression. The colonies were checked regularly for interactions by macroscopic and microscopic analysis. Each interaction was given a score based on mutual intermingling (1-1), mutual antagonism on contact (2-2), mutual antagonism at a distance (3-3), dominance of one species on contact (4-0) and dominance at a distance (5-0). In the case of the dominant interactions, the higher score was always awarded to the more competitive fungus [5]. For example, if *A. carbonarius* was dominant over *B. cinerea* upon contact this would result in a 4 and 0, respectively, being awarded to the two fungal species. The scores for each species were totalled to give an overall Index of Dominancy (I_D_) value as a measure of competitiveness. 

### 4.4. Mycotoxin analyses

*Deoxynivalenol quantification:* The method was adapted from Cooney *et al.* [17]. Grain samples were first dried overnight at 50 °C, milled and a 10 g sub-sample was placed in 40 mL acetonitrile/water (14:1). This was shaken for 2 hr on a rotary shaker before cleanup using an in house cartridge. This cartridge consisted of a 2 mL syringe (Fisher Ltd) packed with a disc of filter paper (Whatman No. 1), a 5 mL lugger of glass wool and 500 mg of alumina/activated carbon (20:1). The sample was allowed to gravity feed through the cartridge. Residues on the cartridge were washed out with acetonitrile/methanol/water (80:5:15; 500 uL). The combined eluate was evaporated to dryness and re-suspended in methanol/water (5:95; 500 uL). 

Quantification of DON was carried out using HPLC using a Luna column (100 mm × 4.6 mm i.d., Phenomonex). Separation was achieved using a isocratic mobile phase of methanol/water (12:88) at 1.5 mL/min. Eluates were detected using a UV detector set at 220 nm and an attenuation of 0.01 AUFS. The retention times for DON was 7.5 min and the limit of quantification for DON was 120 ng/mL. 

*Ochratoxin quantification:* Up to 6 agar plugs (4.5 mm diameter) were removed across the mycotoxigenic strain including the interaction zone. These samples were placed into 28 mL Universal bottles and 5 mL methanol was added. The samples were shaken for 1 hr. The extracts were filtered through fluted filter paper (Whatman No 1) containing 0.25 g of Celite^®^ 545 to improve filtration. This was filtered through a 0.22 um filter (Millex® HV 13mm, Millipore) directly into amber HPLC vials (Jaytee Biosciences LTD, UK) and stored at 4 °C until HPLC analysis was performed. 

The quantification method used was adapted from Bragulat *et al.* [18]. The HPLC system consisted of a Millipore Waters 600E system controller, a Millipore 712 WISP autosampler and a Millipore Waters 470 scanning fluorescence detector (Millipore Corporation Massachusetts USA)(excitation 330 nm, emission 460 nm). The samples were separated using a C18 Luna Spherisorb ODS2 (150 × 4.6 mm, 5 μm) (Pheonomenex), with a guard column of the same material used to extend the column life and reduce drift. Run time for samples was 10 min. The flow rate of the mobile phase (57% acetonitrile, 41% water and 2% acetic acid) was 1 ml/min. The recovery rate for OTA was 88% from grape juice based media with a limit of detection of 0.01µg/g medium. Analysis of the results was carried out on a computer running Kroma system 2000 operating system (Bio-tek Instruments, Milan, Italy). 

## 5. Conclusions

Interactions between mycotoxigenic fungi and other mycobiota are influenced significantly by environmental factors and are thus in a state of flux changing temporally in relation to other stresses such as fungicide/preservative treatments and nutritional quality. Thus the ecological strategies which mycotoxigenic fungi use may differ. Some may use R-selected (Ruderal), C-selected (Combative) or S-selected (Stress) strategies and mycotoxins may be a key component of dominance [3,4]. Sometimes they may use merged secondary strategies (C-R, S-R, C-S, C-S-R) which may include the ability to produce hydrolytic enzymes and the rapid utilization of key carbon sources may then become important in determining niche occupation and exclusion. It may be interesting to study these interactions by examining the key mycotoxin genes involved in the biosynthetic pathway and how their expression may relate to the quantified mycotoxins produced, especially in interaction zones. The availability of microarrays [19] and RT-PCR for key regulatory genes such as the *TRI5* (trichothecene pathway), *FUM1* (fumonisin production) may enable such advances to be now made in understanding the role of mycotoxins in the ecological strategy of different mycotoxigenic species [20,21].

## References

[1] Magan N., Lacey J. (1986). The phylloplane microbial populations of wheat and effect of late fungicide applications. Ann. Appl. Biol..

[2] Bellí N., Mitchell D., Marín S., Alegre I., Ramos A.J., Magan N., Sanchis V. (2005). Ochratoxin A and producing fungi in Spanish wine grapes and their relationship with climatic conditions. Eur. J. Plant. Pathol..

[3] Magan N., Aldred D., Dijksterhuis J., Samson R.A. (2007). Why to fungi produce mycotoxins?. Food Mycology: A Multifaceted Approach to Fungi and Food.

[4] Magan N., Aldred D., van West P., Avery S., Stratford. M. (2007). Environmental fluxes and fungal interactions: maintaining a competitive edge. Stress in Yeasts and Filamentous Fungi.

[5] Magan N., Lacey J. (2004). The effect of water activity, temperature and substrate on interactions between field and storage fungi. Trans. Br. Mycol. Soc..

[6] Marin S., Sanchis V., Ramos A.G., Magan N. (1998). Environmental factors, interspecific interactions, and niche overlap between *Fusarium moniliforme* and *F. proliferatum* and *Fusarium graminearum*, *Aspergillus* and *Penicillium* spp. isolated from maize. Mycol. Res..

[7] Lee H.B., Magan N. (2000). Environmental influences on *in vitro* interspecific interactions between *A. ochraceus* and other maize spoilage fungi on growth and ochratoxin production. Mycopathologia.

[8] Marin S., Magan N., Ramos A.J., Sanchis V. (2004). Fumonisin-producing strains of *Fusarium*: A review of their ecophysiology. J. Food Prot..

[9] Giorni P., Magan N., Battilani P. (2009). Environmental factors modify carbon nutritional patterns and niche overlap between *Aspergillus flavus* and *Fusarium verticillioides* strains from maize. J. Food Microbiol..

[10] Jennings P., Turner J.A., Nicholson P. Overview of *Fusarium* ear blight in the UK-effect of fungicide treatment on disease control and mycotoxin production.

[11] Simpson D.R., Weston G.E., Turner J.A., Jennings P., Nicholson P.  (2001). Differential control of head blight pathogens of wheat by fungicides and consequences for mycotoxin contamination of grain. Eur. J. Plant. Pathol..

[12] Ramakrishna N., Lacey J., Smith J.E. (1996). The effects of fungal competition on colonization of barley grain by *Fusarium sporotrichioides* on T-2 toxin formation. Food Addit. Contam..

[13] Edwards S.C., Pirgozliev S.R., Hare M.C., Jenkinson P. (2001). Quantification of trichothecene-producing *Fusarium* species in harvested grain by competitive PCR to determine efficacies of fungicides against *Fusarium* head blight of winter wheat. Appl. Environ. Microbiol..

[14] Bateman G.L., Murray G. (2001). Seasonal variations in populations of *Fusarium* species in wheat-field soil. Appl. Soil Ecol..

[15] Mitchell D., Parra R., Aldred D., Magan N. (2004). Water and temperature relations of growth and ochratoxin A production by *Aspergillus carbonarius* strainsfrom grapes in Europe and Israel. J. Appl. Microbiol..

[16] De Cal A., Pascual S., Melgarejo P. (1993). Nutritional requirements of antagonists to peach twig blight, *Monilinia laxa*, in relation to biocontrol. Mycopathologia.

[17] Cooney J.M., Lauren D.R., di Menna M.E. (2001). Impact of competitive fungi on trichothecene production by *Fusarium graminearum*. J. Agric. Food Chem..

[18] Bragulat M.R., Abarca M.L., Cabanas F.J. (2001). An easy screening method for fungi producing ochratoxin A in pure culture. Int. J. Food Microbiol..

[19] Schmidt-Heydt M., Geisen R. (2007). A microarray for monitoring the production of mycotoxins in food. Int. J. Food Microbiol..

[20] Jurado M., Marin P., Magan N., González-Jaén M.T. (2008). Relationship between solute and matric potential stress, temperature, and growth and *FUM1* gene expression in two *Fusarium verticillioides* strains from Spai. Appl. Environ. Microbiol..

[21] Doohan F.M., Weston G., Rezanoor H.N., Parry D.W., Nicholson P. (1999). Development and use of a reverse transcription-PCR assay to study the expression of *tri5* by *Fusarium* species *in vitro* and *in planta*. Appl. Environ. Microbiol..

